# Growth Characteristics of *Methanomassiliicoccus luminyensis* and Expression of Methyltransferase Encoding Genes

**DOI:** 10.1155/2017/2756573

**Published:** 2017-11-02

**Authors:** Lena Kröninger, Jacqueline Gottschling, Uwe Deppenmeier

**Affiliations:** Institut für Mikrobiologie und Biotechnologie, Universität Bonn, Bonn, Germany

## Abstract

DNA sequence analysis of the human gut revealed the presence a seventh order of methanogens referred to as Methanomassiliicoccales. *Methanomassiliicoccus luminyensis* is the only member of this order that grows in pure culture. Here, we show that the organism has a doubling time of 1.8 d with methanol + H_2_ and a growth yield of 2.4 g dry weight/mol CH_4_. *M. luminyensis* also uses methylamines + H_2_ (monomethylamine, dimethylamine, and trimethylamine) with doubling times of 2.1–2.3 d. Similar cell yields were obtained with equimolar concentrations of methanol and methylamines with respect to their methyl group contents. The transcript levels of genes encoding proteins involved in substrate utilization indicated increased amounts of mRNA from the *mtaBC2* gene cluster in methanol-grown cells. When methylamines were used as substrates, mRNA of the *mtb/mtt* operon and of the *mtmBC1* cluster were found in high abundance. The transcript level of *mtaC2* was almost identical in methanol- and methylamine-grown cells, indicating that genes for methanol utilization were constitutively expressed in high amounts. The same observation was made with resting cells where methanol always yielded the highest CH_4_ production rate independently from the growth substrate. Hence, *M. luminyensis* is adapted to habitats that provide methanol + H_2_ as substrates.

## 1. Introduction

Among living organisms, methane formation is restricted to methanogenic organisms of the domain *Archaea*. These organisms are important players of the global carbon cycle due to their capability to use fermentation products of organic matter as substrates [1]. While methanogenic archaea have been studied extensively with respect to biogas production and other man-made habitats, little is known about human-associated methanogens [2]. One of these organisms is *Methanomassiliicoccus luminyensis*, which was originally isolated from human feces [3]. This organism is the only member of the seventh order of methanogens, the Methanomassiliicoccales, that is available in pure culture. DNA sequence analysis indicated that members of the Methanomassiliicoccales are also found in gastrointestinal tracts of higher termites and millipedes, the digestive tracts of animals, rice paddy fields, natural wetlands, subseafloor and freshwater sediments [4, 5] and in anaerobic digester sludge [6]. These findings highlight the wide distribution and diversity of members of the order Methanomassiliicoccales which are phylogenetically related to the Thermoplasmatales but only distantly related to other methanogens [4, 7].

Methanogenic archaea are usually divided into three major groups based on their substrate spectrum. While hydrogenotrophic archaea use H_2_ + CO_2_ or formate as substrates, methylotrophic organisms form methane from methylated compounds, and aceticlastic archaea use acetate as substrate [8]. *M. luminyensis* is distinct from these common groups because it uses H_2_ as electron donor but is not able to reduce CO_2_ to CH_4_ [3]. In the first description of the strain, it was stated that methanogenesis is possible by methanol reduction with H_2_ as the electron donor [3]. In this respect, *M. luminyensis* resembled *Methanosphaera stadtmanae*, another methanogenic archaeon that was isolated from human feces and can use methanol in the presence of H_2_ [9, 10]. Genome analysis revealed that besides the genes for methanol utilization, members of the genus *Methanomassiliicoccus* contain complete sets of genes for the degradation of methylamines to CH_4_ and NH_3_ [5, 11, 12]. Lang et al. [12] showed that methane accumulated in the culture headspace of *M. luminyensis* containing methanol, monomethylamine (MMA), dimethylamine (DMA), or trimethylamine (TMA) in the presence of H_2_. In addition, it was found that *M. luminyensis* grows on TMA in the presence of H_2_ but not on TMA in the absence of H_2_ [13, 14]. Due to the ability to use TMA as substrate, *M. luminyensis* is also discussed in the context of the so-called “Archaebiotics” [2, 14]. These therapeutic agents were proposed as a treatment for patients suffering from trimethylaminuria. The patients hold a genetic disposition that prevents oxidation of TMA to trimethylamine oxide (TMAO) in the human liver. TMA is a product of degradation processes in the human gut and can be spread throughout the body by being absorbed from the intestinal lumen and transferred into the bloodstream. When the mechanism to oxidize TMA to TMAO is absent, TMA rapidly accumulates and results in a fish-like odor coming from patients' sweat and breath [15]. The cultivation of *M. luminyensis* in the intestines of patients and the simultaneous metabolization of TMA could reduce the accumulation of TMA in the intestines and could prevent the fish-like odor [14].

Another aspect of “Archaebiotics” based on *M. luminyensis* is the general conversion of TMA into TMAO in the human body. TMAO is thought to raise the risk for developing cardiovascular diseases, for example, arteriosclerosis. Therefore, a low TMAO level is generally desirable in healthy individuals. Due to the fact that *M. luminyensis* produces methane from TMA and TMA oxidation to TMAO does not take place, the occurrence of this archaeon in the human intestinal tract could decrease accumulation of TMAO and consequently the risk for cardiovascular diseases caused by this compound [2].

The metabolism of *M. luminyensis* and members of the Methanomassiliicoccales is unique from other methanogenic archaea because they cannot be classed into the typical methanogenic groups (hydrogenotrophic, aceticlastic, and methylotrophic methanogenic archaea). In fact, *M. luminyensis* is a hybrid of the common methanogenic groups with respect to its mode of energy conservation [11, 12]. When methylated compounds are metabolized by *M. luminyensis*, the methyl group is first transferred by substrate-specific methyltransferases to 2-mercaptoethanol (HS-CoM). In this process, methyl-CoM is formed which is then reduced to methane by the methyl-CoM reductase with 7-mercaptoheptanoyl-threonine phosphate (HS-CoB) as electron donor. This reaction leads to the formation of the heterodisulfide CoM-S-S-CoB. The reduction mechanism of CoM-S-S-CoB to HS-CoM and HS-CoB is not completely resolved in *M. luminyensis*, yet. It is assumed that in the course of the degradation of two molecules of methanol to methane, two molecules of heterodisulfide are formed [11, 12]. One of the heterodisulfide molecules is reduced by a multienzyme complex consisting of a [NiFe] hydrogenase (Mvh) and a heterodisulfide reductase (HdrABC), which uses H_2_ as electron donor and transfers electrons to heterodisulfide and ferredoxin (Fd) in a bifurcation reaction. It was suggested that Fd_red_ can then be oxidized by a membrane-bound dehydrogenase (Fpo complex), which is similar to the H^+^-translocating NADH dehydrogenase (complex 1) found in the respiratory chain of eukaryotes and many bacteria. In *M. luminyensis*, a second heterodisulfide reductase (HdrD) serves as electron-accepting unit and reduces the second heterodisulfide molecule [11, 12]. During Fd_red_ oxidation and simultaneous heterodisulfide reduction, protons can be translocated across the membrane to establish an electrochemical gradient, which is needed for ATP synthesis.

Because of the importance of *M. luminyensis* with respect to the human gut microbiome and its interesting mode of CH_4_ formation, it is essential to analyze the physiology and biochemistry of this organism. Here, we present data on the growth behavior and gene expression of *M. luminyensis* for the development of methods that permit efficient growth and allow the production of sufficient cell mass for further biochemical analysis of the metabolism of this organism.

## 2. Materials and Methods

### 2.1. Strains and Culture Conditions

*M. luminyensis* DSM 25720 was obtained from the Deutsche Sammlung für Mikroorganismen und Zellkulturen (Braunschweig, Germany). Cultures (50 or 500 mL) were grown in complex medium as described by Hippe et al. [16] under a hydrogen atmosphere (200 kPa). Methanol, MMA, DMA, and TMA were added prior to inoculation as indicated from sterile stock solutions. Na_2_S (0.3 g/L) and cysteine (0.9 g/L) were added as reducing agents. Ampicillin (100 *μ*g/mL) and tetracycline (10 *μ*g/mL) were applied to ensure culture purity. Cells were grown at 37°C without agitation, and growth was monitored by measuring the optical density at 600 nm.

### 2.2. Quantification of CH_4_, Methanol, MMA, DMA, TMA, and Acetate

CH_4_ and methanol contents were quantified using gas chromatography (GC), respectively. For CH_4_ quantification, 25 *μ*L samples were taken from the headspace of the cultures and injected into a GC (PerkinElmer Clarus® 480, Rascon FFAP column 25 m × 0.25 micron, PerkinElmer, Waltham, USA) with a flame ionization detector (FID). Measurements were conducted at a column temperature of 120°C, an injector temperature of 150°C, and a detector temperature of 250°C. N_2_ was used as carrier gas. A CH_4_ standard with a defined concentration of 10% (90% argon) was used as reference and was measured prior to and during measurement series.

For methanol quantification, 1 *μ*L samples from culture supernatants were injected into a GC (Shimadzu GC-14A, Shimadzu, Duisburg, Germany with Agilent Chromosorb 101 column, Agilent Technologies, Santa Clara, USA) with a column temperature of 100°C, an injector temperature of 220°C and a detector temperature of 220°C with N_2_ as carrier gas. The methanol content in the culture supernatants was quantified by correlation to a methanol standard curve.

For MMA, DMA, and TMA quantification, the cells were separated from the medium by centrifugation. To remove Na_2_S from the samples, the supernatant was mixed with H_2_O_2_ (0.3% (*v/v*) final concentration). The precipitate was removed by centrifugation (20,000 ×g, 1 min), and the cleared supernatant was used for methylamine quantification. All further steps were performed as described by Krätzer et al. [17]. Acetate was quantified by HPLC on an Aminex HPX-87H (BioRad, Munich, Germany, 300 mm × 7.8 mm) column at 25°C. 5 mM H_2_SO_4_ was used as eluent, and a flow rate of 0.3 mL/min was applied. Samples were prepared by adding 300 *μ*L 5 mM H_2_SO_4_ to 300 *μ*L of the culture supernatant. The samples were centrifuged to remove precipitates and were filtered. 20 *μ*L of the mixture was then applied to HPLC. Standard curves were prepared for all compounds from 0 to 50 mM concentrations.

### 2.3. Determination of Dry Weight

For dry weight determination, 500 mL cultures of *M. luminyensis* were inoculated as mentioned above. At each time point, the optical density was recorded and 50 mL samples were withdrawn from the culture. The cells were harvested and washed by centrifugation (6000 ×g, 20 min, 10°C). The pellets were dried at 70°C and weighed after two days.

### 2.4. qRT-PCR

Total RNA from *M. luminyensis* was obtained from 500 mL cultures grown to mid exponential phase on 150 mM methanol + H_2_, 50 mM TMA + H_2_, 75 mM MMA + H_2_, or 75 mM DMA + H_2_. Cells were harvested by centrifugation (11,000 ×g, 15 min, 4°C) and resuspended in 3 mL TRI reagent (Sigma-Aldrich, Steinheim, Germany) for RNA stabilization and cell lysis. RNA was extracted using 1-bromo-3-chloropropane and was purified by RNA Clean & Concentrator™-5 kit (Zymo Research, Freiburg, Germany). DNAse I was applied to RNA samples to remove DNA contaminations. RNA concentration and purity were determined spectrophotometrically using a BioSpectrometer® according to a standard protocol (Eppendorf, Hamburg, Germany). Control PCR experiments confirmed that the RNA samples did not contain DNA contaminations. The primer sequences that target genes coding for substrate-specific corrinoid proteins are shown in Table 1. Homologous genes were aligned using the program Clustal Omega [18], and regions showing maximal differences were chosen for primer generation. The ribosomal S15 gene (WP_026068997) was used as reference gene. To analyze cross-hybridization between highly homologous corrinoid protein-encoding genes, we checked annealing of primer combinations of different homologous genes under real-time PCR conditions using chromosomal DNA as templates. It came out that only combinations of specific forward and reverse primers designed for each gene were efficient in PCR and in qRT-PCR, indicating that there was no significant cross-annealing between homologous genes. qRT-PCR reactions were performed using a qRT-PCR Green Mix One Step Kit (biotechrabbit, Hennigsdorf, Germany) which is based on SYBR Green as fluorescence dye. To each PCR reaction, 150–200 ng RNA was applied and a negative control without reverse transcriptase was carried along to verify that DNA contaminations did not affect the results. To perform the PCR steps and to measure fluorescence, the cycler CFX Connect™ and a suitable software (BioRad, Munich, Germany) were used. The PCR fragments resulted in a single peak in the melting curve analysis, respectively. For calculations of ΔCt values, the quantification cycle (Ct) of the reference gene (ribosomal protein 15S) was subtracted from the Ct value of each gene of interest. The fold change of each gene was calculated using the formula 2−^ΔCt^.

### 2.5. Preparation of Resting Cells

Resting cells were prepared from cultures grown on 150 mM methanol + H_2_ or 50 mM TMA + H_2_ as described above. Cultures were grown to mid log phase (OD_600_ 0.1-0.2) and harvested by centrifugation (11,000 ×g, 15 min, 10°C). The cell pellets were washed twice using stabilization buffer (17 mM NaCl, 7 mM KCl, 2 mM MgCl_2_ × 6 H_2_O, 1 mM CaCl_2_ × 2 H_2_O, 6 mM NH_4_Cl, 1.5 mM KH_2_PO_4_, 1 mM Na_2_SO_4_, 20 mM BisTris, and 200 mM sucrose and pH 7.4) and finally resuspended in stabilization buffer. 2 mL of the resting cell suspension was filled into a 9 mL rubber stoppered glass vial flushed with H_2_. Cells were equilibrated at 37°C and 200 rpm for 5 min. Methane production was initiated by the addition of a methyl group donor (75 mM methanol, 75 mM MMA, 37.5 mM DMA, or 25 mM TMA) from anaerobic stock solutions. During the experiment, the cells were incubated at 37°C and 200 rpm. Samples from the headspace (25 *μ*L) were taken at different time points and analyzed by gas chromatography as described above to monitor CH_4_ production.

## 3. Results

### 3.1. Growth on Methanol

**Figure 1:**
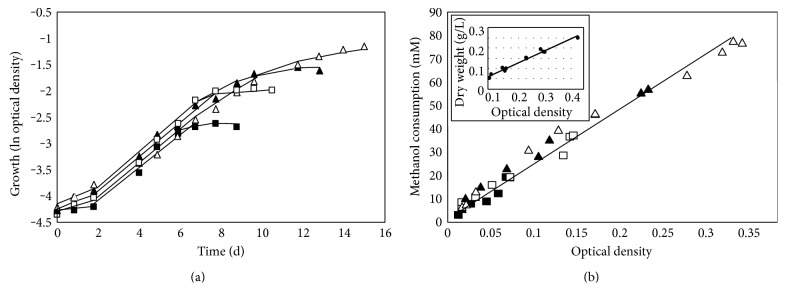
Growth of *M. luminyensis* cultures in media containing different amounts of methanol. (a) Growth on media containing 20 mM methanol (black squares), 40 mM methanol (white squares), 60 mM methanol (black triangles), or 80 mM methanol (white triangles). (b) Colinearity between optical density and methanol consumption. Media contained 20 mM methanol (black squares), 40 mM methanol (white squares), 60 mM methanol (black triangles), or 80 mM methanol (white triangles). Inset: correlation between optical density and dry weight.

There is still very little known about the physiology and biochemistry of members of the order Methanomassiliicoccales and the enzymes, which are involved in the methanogenic pathway. Since the methanol concentration for optimal growth of *M. luminyensis* was not analyzed in detail yet, the organism was grown in complex media containing different amounts of methanol under a H_2_ atmosphere (200 kPa). Growth of the cells was examined after addition of 20, 40, 60, or 80 mM methanol (Figure 1). Samples from the culture medium were used to analyze optical density, cell dry weight, and methanol concentration. All cultures showed a lag phase of about 2 d followed by an exponential phase of 5–10 d depending on the methanol content. The growth rate of 0.38 ± 0.04 d^−1^ was almost identical in all cultures independently from the amount of methanol that was added (Figure 1(a)). The corresponding doubling time (t_d_) was 1.8 ± 0.2 d. Gas chromatographic analysis of the culture supernatant indicated an exponential increase in methanol consumption that directly correlated with the optical density. Methanol was almost completely consumed at the beginning of the stationary growth phase (not shown). The time point of substrate exhaustion depended on the methanol concentration at the beginning of the experiment. Hence, growth of the cells stopped after 6.5 d (20 mM methanol), 8 days (40 mM methanol), 9.5 d (60 mM methanol), and 12 d (80 mM methanol), respectively (Figure 1(a)). Further evidence that substrate utilization was directly coupled to the increase of cell mass of the cultures resulted from the correlation of methanol consumption and optical density in the course of the exponential growth phase (Figure 1(b)). From the slope of the curve, it was calculated that *M. luminyensis* had to convert 24 ± 2 mM methanol to reach an optical density of 0.1. In addition, it became evident that there was a linear correlation between optical density and dry weight (optical density of 1 = 0.59 g dry weight/L; Figure 1(b), inset). From these data, a growth yield of 2.4 g dry weight/mol methanol could be calculated.

**Figure 2:**
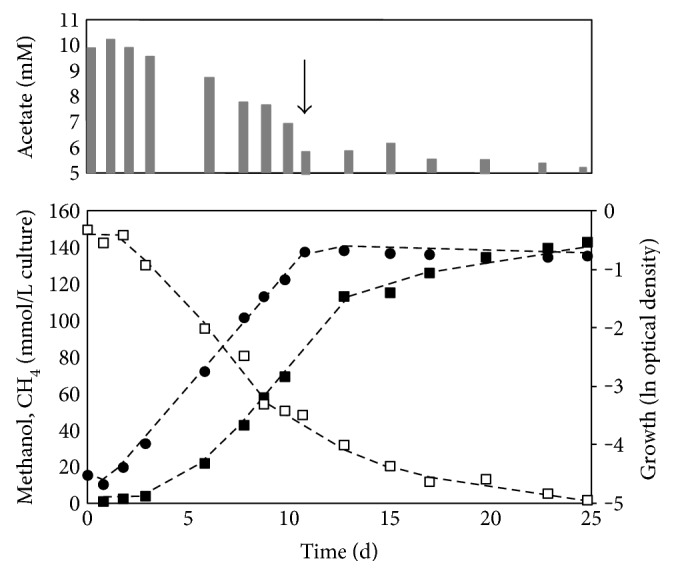
Growth parameters and acetate consumption during growth in media containing 150 mM methanol. Methanol concentration (white squares), CH_4_ concentration (black squares), ln optical density (black circles), and acetate concentration (gray bars). Black arrow indicates the beginning of the stationary phase. The methanol/methane ratio was calculated from the slopes of the methanol consumption rate and the CH_4_ formation rate in the exponential growth phase.

Methanol consumption and methane formation were not measured simultaneously, yet. To check the stoichiometry, *M. luminyensis* was grown under a H_2_ atmosphere at 37°C in complex medium containing 150 mM methanol. Samples from the gas phase were frequently taken to quantify the methane content in the headspace. Furthermore, samples from the culture medium were used to analyze the methanol concentration. It was found that 150 mM methanol was degraded within 25 d and equimolar amounts of CH_4_ were formed (Figure 2). All samples taken from different cultures and at different time points indicated that methanol and methane were formed in a ratio of 0.98 ± 0.2. The doubling time during growth on 150 mM methanol was 1.9 ± 0.1 days. Additionally, the methane production rate (mmol CH_4_ L culture^−1^ d^−1^) of the cultures was analyzed. The rate strongly correlated with the optical density and the growth phase of the cultures. The highest methane production rate was observed at the end of the exponential phase and reached a value of 14 mmol CH_4_ L culture^−1^ d^−1^ (not shown). Once the stationary phase was reached (day 10), the methane production rate decreased sharply and in the end, CH_4_ formation almost stopped completely. Interestingly, methanol was not the limiting growth factor because the stationary phase started (day 10) before methanol was completely utilized. A detailed analysis revealed that the cultures reached a maximal optical density of 0.5 and degraded only about 110 mM methanol in the course of the exponential phase (more details are shown in Figure S1 available online at https://doi.org/10.1155/2017/2756573). The remaining substrate was slowly consumed over one week during stationary phase without further cell growth (Figure 2). H_2_ was not a limiting factor since this gas was always added in exceeding amounts by gassing the cultures after each sampling. In addition, the separate addition of double amounts of salts, acetate, vitamins, trace elements (including tungsten and selenite [3]), iron ions, amino acids, yeast extract, or yeast/peptone did not result in an increase of the optical density. Furthermore, the complementation of media with rumen fluid, fluid from biogas plant sludge, cell-free extracts from *Methanosarcina mazei* or *Methanotorris igneus*, or short-chain fatty acids had no effect on the growth efficiency. Hence, the reason why growth of *M. luminyensis* stopped after the consumption of about 110 mM methanol is unknown.

We also tested whether acetate as component of the complex medium could be used as carbon source. HPLC analysis of cell-free culture supernatant indicated that the acetate concentration decreased from 10.2 mM to 5.5 mM in the course of the exponential phase (Figure 2). A detailed analysis of acetate degradation and cell growth indicated that 16.5 ± 2 mmol acetate (or 33 mmol carbon atoms) was consumed for the formation of 1 g dry weight (Figure S2). Assuming that the dry mass of *M. luminyensis* contains 47% carbon [19], the total amount of carbon sums up to 39.2 mmol per g dry weight. Hence, about 84% of cellular carbon derived from acetate.

### 3.2. Growth on Methylamines

**Figure 3:**
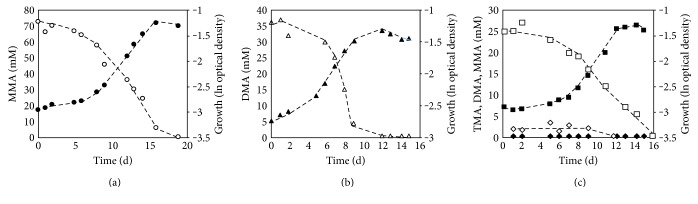
Growth of *M. luminyensis* on methylamines. The organism was grown in the presence of H_2_ (200 kPa). (a) MMA + H_2_: MMA concentration (white circles) and ln optical density during growth on MMA (black circles). (b) DMA + H_2_: DMA concentration (white triangles) and ln optical density during growth on DMA (black triangles). (c) TMA + H_2_: optical density (black squares), TMA concentration (white squares), DMA concentration (white diamonds), and MMA concentration (black diamonds).

To analyze methylamine degradation in detail, *M. luminyensis* was cultured in complex medium containing different methylamines under a H_2_ atmosphere (200 kPa) and several growth parameters were determined (Figure 3). 75 mM MMA was completely degraded in the presence of H_2_ in the headspace of the cultures within 18 d (Figure 3(a)). The utilization of MMA coincided with the increase of the optical density. The final optical density of 0.3 was in the same range as with 75 mM methanol as substrate. The doubling time in the exponential phase was 2.3 ± 0.5 d and was longer in comparison to growth on methanol. MMA was almost entirely consumed at the end of the exponential phase.

To investigate growth of *M. luminyensis* on DMA, the organism was grown in the presence of 37.5 mM DMA + H_2_ and the optical density, as well as the DMA concentration in the medium were monitored over time (Figure 3(b)). DMA possesses two methyl groups for methanogenesis. Therefore, only half of the DMA concentration was needed in comparison to MMA or methanol to obtain the same growth yield. After inoculation of the medium, DMA was successively degraded while the optical density of the culture increased simultaneously. The cells grew with a growth rate of 0.32 ± 0.07 d^−1^ and a doubling time of 2.2 ± 0.5 d. When DMA was completely consumed, the organism stopped growing and merged into stationary phase (Figure 3(b)). At this time point, the optical density reached 0.28 which was in good agreement with the final optical density obtained with 75 mM MMA + H_2_.

TMA contains three methyl groups, which are sequentially converted into methane in *Methanosarcina* species [17, 20]. Hence, DMA and MMA are possible intermediates of TMA degradation and could accumulate in the medium. To investigate TMA utilization in *M. luminyensis*, substrate consumption, product formation, and growth parameters were analyzed (Figure 3(c)). The TMA concentration decreased continuously until almost all substrate molecules were consumed. The final optical density of cultures grown on 25 mM TMA was 0.28. This value was in the same range of the final optical densities of cultures grown on 75 mM methanol or 75 mM MMA, respectively. Due to the fact that TMA possesses three methyl groups, a TMA concentration of 25 mM equals a MMA (or methanol) concentration of 75 mM with respect to the amount of methyl group equivalents per substrate molecule. TMA-degrading cultures showed a doubling time of 2.1 ± 0.4 d which was the fastest growth rate observed for methylamines. Interestingly, at the beginning of the exponential growth phase, significant concentrations of DMA could be detected in the culture supernatants reaching concentrations of up to 4 mM. In the late exponential phase, the entire amount of DMA was consumed (Figure 3(c)). In contrast to *Ms. mazei* [17], *M. luminyensis* did not form extracellular MMA as an intermediate of methanogenesis from TMA. To analyze the efficiency of the energy conserving system of *M. luminyensis* grown on TMA in comparison to methanol, the growth yield was determined for cultures growing on TMA. Interestingly, the same growth yield of 2.4 g dry weight/mol CH_4_ was determined for TMA and methanol suggesting that both substrates are equally suitable substrates for the overall metabolism.

### 3.3. Analysis of the Transcript Level of Genes Encoding Enzymes Involved in Substrate Demethylation

**Figure 4:**
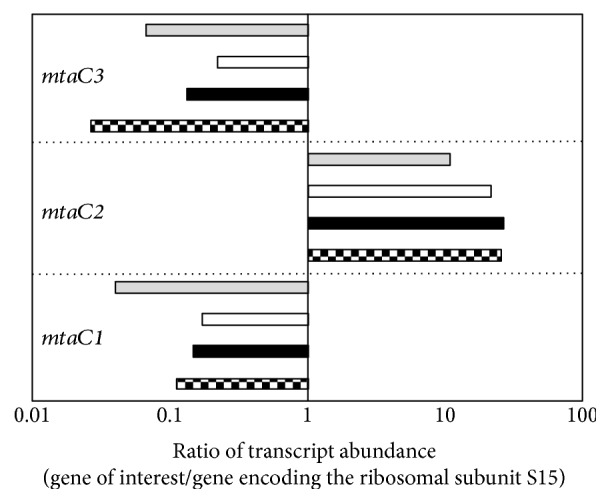
mRNA abundance of genes encoding corrinoid proteins responsible for methanol degradation (*mtaC1–3*) in *M. luminyensis* during growth on methanol (checked bars), TMA (gray bars), DMA (white bars), or MMA (black bars). ΔCt values were calculated using the relative amount of ribosomal protein S15 transcripts as reference (C_T_ gene—C_T_ S15). Ratios were calculated from ΔCt values using the function 2^−ΔCt^. The cells were harvested from three different cultures in the mid exponential phase (average optical density = 0.15).

**Figure 5:**
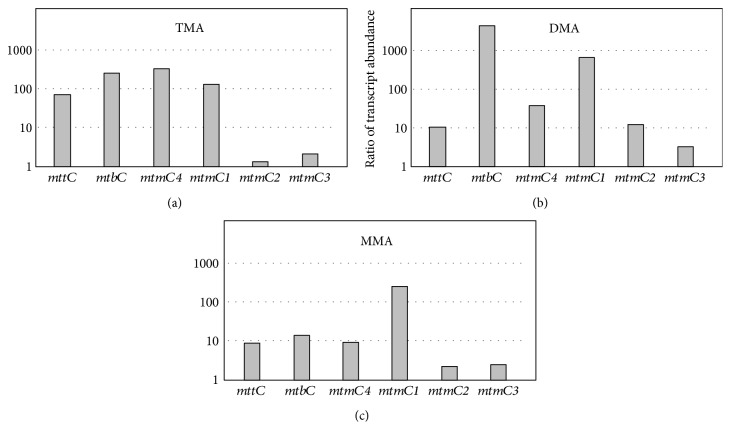
Ratios of transcript abundances of genes encoding corrinoid proteins involved in the degradation of methylamines in *M. luminyensis*. The abundance of a certain gene transcript was compared to its abundance during growth on methanol. ΔC_T_ values were calculated using the relative amount of reference gene transcripts as a standard (C_T_ gene—C_T_ S15). ΔΔCT values derived from the ΔCT values of genes of interest in methanol-grown cultures minus the ΔCT values of genes of interest in TMA- (a), DMA- (b), or MMA- (c) grown cultures. Ratios were calculated from ΔΔC_T_ values by using the function 2^ΔΔCT^.

Methylated C1 substrates are channeled into the methylotrophic pathway of methanogenesis by substrate-specific methyltransferases [21]. To analyze which genes are involved in consumption of methanol or the three methylamines, qRT-PCR experiments were performed using gene-specific primers and RNA preparations from *M. luminyensis* cultures grown on the respective substrate and harvested in the mid log phase. Primers were designed for the specific amplification of transcripts from nine different genes encoding corrinoid-containing methyl-accepting proteins which are predicted to participate in methyl group transfer from the substrates to HS-CoM. These corrinoid proteins are substrate specific and are referred to as MtaC, MttC, MtbC, and MtmC for the breakdown of methanol, TMA, DMA, and MMA, respectively. The corresponding genes are found on the chromosome of *M. luminyensis* in one to four copies [7, 12]. Single genes are present for TMA (*mttC*) and DMA (*mtbC*) utilization, respectively. The corrinoid protein involved in methanol consumption is encoded by three different genes (*mtaC1*, *mtaC2*, and *mtaC3*). Four copies of the gene *mtmC* (*mtmC1*, *mtmC2*, *mtmC3*, and *mtmC4*) were detected encoding the corrinoid protein MtmC for MMA degradation. In addition, primers were generated for the gene encoding the ribosomal subunit S15 which was used as a reference in the qRT-PCR studies to determine expression levels. It is most likely that this gene is highly expressed during the exponential growth phase. The qRT-PCR results indicated different transcription patterns when the cells were grown on methanol and methylamines, respectively (Figures 4 and 5). During growth on methanol, the gene *mtaC2* showed a 15-fold higher mRNA concentrations in comparison to the reference gene encoding the ribosomal protein S15, indicating the importance of the corresponding corrinoid protein for methanol metabolism (Figure 4). In contrast, *mtaC1* and *mtaC3* were transcribed at low levels with 10- to 50-fold lower transcript abundance in comparison to the reference gene. The close association of *mtaB* and *mtaC* genes in the genome suggests that they form transcription units as in other methanogens [17, 22]. Hence, the results indicate that methyl group transfer from methanol is mainly performed by the corrinoid protein MtaC2 in connection with the methyltransferase MtaB2. Gene transcripts encoding corrinoid proteins for methylamine utilization (*mttC*, *mtbC*, and *mtmC1–C4*) were only found in low amounts during methanogenesis from methanol (Supplementary Table 1 available online at https://doi.org/10.1155/2017/2756573). This expression pattern considerably changed during growth on TMA. The genes *mttC* and *mtbC* revealed a 75- and 200-fold higher expression value in TMA-grown cells compared to cells grown on methanol, respectively (Figure 5(a)). Therefore, the corresponding corrinoid proteins together with their partners MttB and MtbC are probably involved in the first and second methyl group transfer from TMA to HS-CoM. With respect to MMA utilization during growth on TMA, it became evident that *mtmC4* had the highest transcript concentration of all *mtmC* genes followed by *mtmC1* (Figure 5(a)). In detail, we observed a 130 and a 325-fold increase of *mtmC1* and *mtmC4* transcripts with TMA as substrate in comparison to methanol grown cells, respectively. The genes *mtmC2* and *mtmC3* showed a very low transcript abundance in TMA cells and a negligible increase of mRNA concentration compared to methanol grown cells (Figure 5(a)). Hence, we assume that *mtmC1* and *mtmC4* encoding corrinoid proteins and their corresponding MT1 enzymes (MtmB1 and MtmB4) are of major importance for the degradation of the intracellular intermediate MMA during growth on TMA. The genes *mtbC* and *mtmC1* were also highly expressed when the cells were grown on DMA whereas the abundance of *mtmC4* and *mttC* gene transcripts decreased (Figure 5(b)). When MMA was the substrate, only *mtmC1* showed a high transcript concentration (Figure 5(c)). Interestingly during growth on methylamines, *mtaC* genes were expressed to almost the same extent as in cells grown on methanol (Figure 4). In summary, qRT-PCR analysis of key genes involved in methyl group transfer during methanogenesis led to the conclusion that *M. luminyensis* is able to adapt to TMA, DMA, and MMA as substrates, while the transcript levels of *mtaC* genes involved in methanol utilization were virtually unchanged. Hence, we assume that the organism prefers methanol as substrate and possesses a regulatory network for methylamine utilization.

### 3.4. Methane Formation in Resting Cells

**Figure 6:**
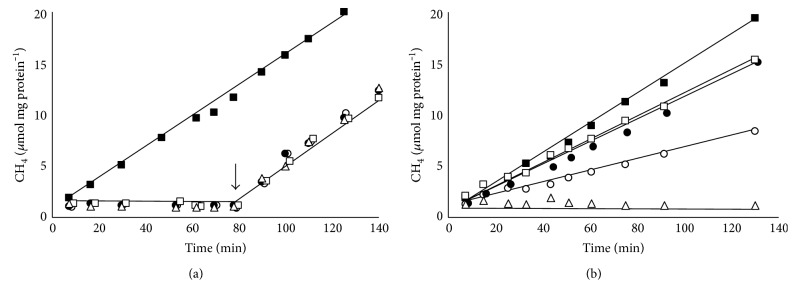
Resting cell experiments with methanol- or TMA-adapted cells. (a) Methanol-adapted resting cells fed with methanol (black squares), TMA (black circles), DMA (white squares), MMA (white circles), or without substrate (white triangles). The black arrow indicated the addition of 75 mM methanol. (b) TMA-adapted cells supplemented with methanol (black squares), TMA (black circles), DMA (white squares), MMA (white circles), or without substrate (white triangles).

Evidence in favor for the hypothesis that methanol is the preferred substrate came from experiments with resting cells. When resting cells obtained from methanol-grown cultures were incubated with methanol + H_2_ as substrate, CH_4_ was formed with a rate of 164 nmol min^−1^ mg protein^−1^ (Figure 6(a)). Interestingly, there was almost no methane production from TMA + H_2_, DMA + H_2_, or MMA + H_2_. A control experiment showed that the cells were not inactive but could produce methane in the same range of methanol-grown cells when methanol was added after 80 min (Figure 6(a)). This observation fits to the finding that transcripts of genes encoding corrinoid proteins for the degradation of methylamines were only found in very low amounts during growth on methanol (Table S1) whereas transcripts of genes for methanol degradation were present in abundance. Similar experiments were performed with resting cells from TMA-grown cultures (Figure 6(b)). In this case, methane formation rates from TMA + H_2_ (150 nmol min^−1^ mg^−1^), DMA + H_2_ (153 nmol min^−1^ mg^−1^), or methanol + H_2_ (177 nmol min^−1^ mg^−1^) were in the same range compared to cells from methanol-grown cultures that converted methanol + H_2_. Only methanogenesis from MMA was a little slower with rates of 59 nmol min^−1^ mg^−1^. The experiments clearly showed that CH_4_ formation from methylamines was only possible when cultures were grown with TMA as substrate. These findings were in line with the results from qRT-PCR experiments which indicated that the genes involved in methylamine degradation were only transcribed to a sufficient extent during growth on TMA. Methanol + H_2_ was always consumed irrespective whether the cells were grown on methanol or TMA.

## 4. Discussion

The order Methanomassiliicoccales is diverse, and members are found in habitats, which are highly relevant for global methane production. *M. luminyensis* was isolated from human feces and could evolve into a model organism of the order Methanomassiliicoccales; because the genome sequence is known, it is culturable in the laboratory and may have impacts on the human gut and on the biogas formation process [7]. *M. luminyensis* showed the best growth rate with methanol + H_2_ as substrates. The final optical density strongly correlated with the methanol content, and the amount of methane increased proportionally. The highest final optical density was obtained with 110 mM methanol. Higher methanol concentrations did not lead to an increased final optical density. When methylamines were added in equimolar concentrations in reference to their methyl group content, similar final optical densities were obtained with 75 mM methanol, 75 mM MMA, 37.5 mM DMA, and 25 mM TMA as substrate, respectively. While the final optical densities of cultures growing on these substrates were in good agreement, the doubling times slightly varied from 1.8 d with methanol to 2.3 d with MMA.

A common method to compare methanogenic archaea with respect to the efficiency of their energy conserving systems is the determination of growth yields. The growth yield is defined as the amount of dry cell mass (g) which is obtained per mole of methane that is formed and is referred to as Y_CH4_. Y_CH4_ strongly correlates with the mode of energy conservation in methanogenic archaea. Hydrogenotrophic methanogens that do not possess cytochromes are only able to produce comparably low amounts of cell mass during methane formation, while archaea with cytochromes, for example, the genus *Methanosarcina*, produce higher cell mass under comparable conditions (Table 2). This observation argues for the hypothesis that the overall ATP gain is smaller in hydrogenotrophic methanogens with only 0.5 molecule of ATP compared to cytochrome containing methanogens which synthesize 1 molecule ATP per molecule methane [23, 24]. Here, the growth yield was determined for *M. luminyensis* on methanol + H_2_ and TMA + H_2_ which resembled the growth yield of hydrogenotrophic methanogens with 2.4 g of cell dry mass being formed per mole methane, respectively. This finding leads to the hypothesis that the ATP yield in *M. luminyensis* is comparable to the yield of hydrogenotrophic representatives with only 0.5 ATP molecules being formed during the generation of one molecule of methane. This is in good agreement with the metabolic model that was previously proposed for *M. luminyensis* [11, 12], where the translocation of 3-4 protons per two molecules of CH_4_ was predicted.

**Figure 7:**
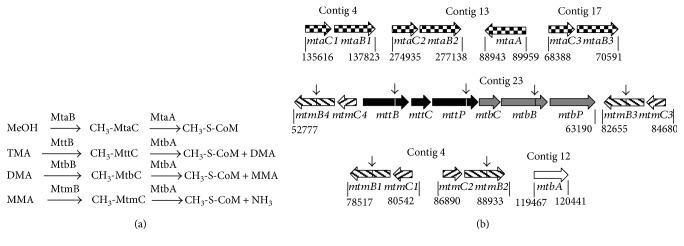
Gene clusters encoding enzymes for methyl group transfer to HS-CoM. (a) Methyl group transfer reactions. (b) Gene clusters encoding enzyme for methyl group transfer. The size of the arrows represents the relative length of the genes. Vertical black arrows indicate the position of the pyrrolysine encoding codon UAG. Genes involved in TMA, DMA, MMA, and methanol degradation are indicated by black, gray, dashed, and dotted vertical arrows, respectively. White arrows represent genes encoding general enzymes involved in methylamine utilization. Numbers show the beginning and the end of the gene clusters, respectively. For details, see Borrel et al. [5]. *mtaC1*, WP_019176359.1; *mtaB1*, WP_026068687.1; *mtaC2*, WP_019177725.1; *mtaB2*, WP_019177726.1; *mtaC3*, WP_026069014.1; *mtaB3*, WP_019178088.1; *mtaA*, WP_026068914.1; *mtbA*, WP_019176765.1; *mtmB4*-C-term, WP_019178516; *mtmB4*-N-term, WP_026069098; *mtmC4*, WP_019178518; *mttB*-N-term, WP_026069099.1; *mttB*-C-term, WP_049796374.1; *mttC*, WP_026069100.1; *mttP*-N-term, WP_049796368.1; *mttP*-C-term, WP_019178523.1; *mtbC*, WP_026069102.1; *mtbB*-N-term, WP_026069103.1; *mtbB*-C-term, WP_026069104.1; *mtbP*, WP_049796369.1; *mtmB1*-C-term, WP_019176305.1; *mtmB1*-N-term, WP_019176306.1; *mtmC1*, WP_026068678.1; *mtmC2*, WP_026068679.1; *mtmB2*-N-term, WP_019176314.1; *mtmB2*-C-term, WP_019176315.1; *mtmB3*-C-term, WP_019178553; *mtmB3*-N-term, WP_019178554; *mtmC3*, WP_049796371. Not shown: putative MMA transporter with low homology to the corresponding enzyme from *Ms. mazei* [16], WP_01976434 (contig 5, 35060-37315).

It was demonstrated that *Methanosarcina* species transfer methyl moieties from their substrates methanol or methylamines to HS-CoM forming methyl-coenzyme M (methyl-CoM) as central intermediate in methanogenesis [31]. These reactions are catalyzed by the combined activities of methyltransferase 1 (MT1) [32, 33], a corrinoid protein and methyltransferase 2 (MT2) [34–36]. MT1 proteins are encoded by the genes *mtaB* and *mttB*, for methanol and TMA degradation, respectively (Figure 7) [37]. Furthermore, *mtbB* and *mtmB* genes encode MT1 enzymes for the utilization of DMA and MMA [20, 38]. The enzymes transfer the methyl group from the substrates to the corresponding corrinoid proteins that are encoded by the genes *mtaC* for methanol, *mttC* for TMA, *mtbC* for DMA, and *mtmC* for MMA. Methyl moieties bound to the corrinoid proteins are transferred to HS-CoM by MT2 proteins (Figure 7) [33]. Here, two classes are known which either use methylated MttC, MtbC, or MtmC proteins (MtbA) or MtaC as substrates (MtaA). However, it was found that MtaA is also involved in the transfer of the methyl group originating from TMA, albeit less efficiently [33, 39]. The final product from the methyl group transfer is methyl-CoM that is reduced to methane by the methyl-CoM reductase using coenzyme B (HS-CoB) as reductant.

The genome sequence of *M. luminyensis* indicated that several clusters comprise genes, which encode methyltransferases involved in methane formation from methanol and methylamines, respectively [5, 12]. For the demethylation of methanol, three copies of genes for MT1 (*mtaB1*, *2*, and *3*), the corrinoid protein (*mtaC1*, *2*, and *3*), and one gene encoding the methanol-specific MT2 (*mtaA*) were identified (Figure 7). The gene clusters for methylamine utilization are even more complex. The largest cluster was found on contig 23 of the genome, which contained a full set of genes involved in DMA (*mtbBC*) and TMA (*mttBC*) degradation, including genes encoding permeases for TMA (*mttP*) and DMA (*mtbP*) uptake. Furthermore, genes encoding enzymes for the synthesis of pyrrolysine (*pylBCD*) and tRNA^Pyl^ (*pylS*) were identified (not shown) [5]. While *mtbBC* and *mttBC* were found in single copies, four clusters for MMA utilization (*mtmBC*) were detected. One cluster on contig 23 was located next to the *mtt*/*mtb*/*pyl locus* but was encoded on the complementary DNA strand (Figure 7). Two copies were found on contig 4, which were separated by a second *pylBCD*/*pylS* cluster. The fourth *mtmBC* gene cluster on contig 23 was connected to a third *pylC* gene and a gene encoding the nitrogen regulator PII (not shown) [5].

Interestingly, all genes encoding MT1 enzyme for methylamine degradation contain the amber codon UAG. Hence, all methylamine corrinoid methyltransferases (MttB, MtbB, and MtmB) possess the 22nd proteogenic amino acid pyrrolysine [5]. It is synthesized by PylBCD from two lysine molecules and bound to a Pyl-specific tRNA by the tRNA synthetase PylS for incorporation via the amber codon (UAG) [21]. Such a system has been found only in the methanogens of the family Methanosarcinaceae and in a small number of bacteria.

From our results, it became evident that *M. luminyensis* adapts to different methanogenic substrates. qRT-PCR analysis revealed differences in mRNA abundances of genes encoding the enzymes for the utilization of methanol and methylamines, respectively. An interesting aspect was the fact that only one (*mtaC2*) out of the three *mtaC* genes was highly expressed. Similar results were obtained for *Methanosarcina* species where qRT-PCR, proteome analyses, and reporter gene fusion assays, as well as deletion mutant studies, were performed to investigate the role of the different methyltransferase systems in the degradation of methanol [37, 40–43]. It is tempting to speculate that *mtaC2* in *M. luminyensis* forms an operon structure with *mtaB2*. Evidence for this presumption was found in *Ms. barkeri* and *Ms. mazei* where *mtaBC* genes are cotranscribed [17, 22]. Therefore, it can be concluded that in *M. luminyensis* methyl group, transfer from methanol to the corrinoid protein MtaC2 is catalyzed by MtaB2. The methyl group of CH_3_-MtaC2 is then transferred to coenzyme M by the catalytic activity of the MT2 methyltransferase MtaA. Furthermore, qRT-PCR experiments showed increased mRNA levels of the *mttC* and *mtbC* genes in TMA-grown cultures reaching ratios up to 300-fold of those in methanol-grown cells. The genes *mttC* and *mtbC* are part of a large gene cluster containing the corresponding *mttB* and *mtbB* genes as well as genes encoding potential permeases for TMA and DMA uptake, respectively (Figure 7). Hence, all results point to the presence of a highly elaborate mechanism of gene regulation for optimal TMA utilization in *M. luminyensis*. In addition, it became evident that the *mtaC* genes were not differentially expressed and especially *mtaC2* revealed high expression levels during growth on methanol and all methylamines, respectively. These findings were also underlined by experiments with resting cells. Cells, which were grown on methanol, could produce CH_4_ from methanol + H_2_ but not from methylamines + H_2_ suggesting that genes involved in methylamine degradation are stringently repressed during growth on methanol. In contrast, resting cells could use methylamines as substrates when they were adapted to and grown on TMA. However, in this case, methanol still yielded the highest methane production rate, which argues for the hypothesis that genes for methanol degradation are constitutively expressed and highly transcribed independently from the substrate.

## 5. Conclusion

Information about growth parameters of *M. luminyensis* and other human-associated methanogens are scarce, although *M. luminyensis* could evolve into a suitable model organism for studying the biochemistry of methanogenic archaea from the order Methanomassiliicoccales and the biology of human-associated archaea in general. Furthermore, for a successful development of curative treatments (“Archaebiotics”) using *M. luminyensis*, it is essential to analyze the growth behavior for the development of methods that permit efficient growth and allow the production of sufficient cell mass. In recent studies, only low cell yields were obtained with *M. luminyensis* [14]. Here, we present comprehensive data about how growth of this organism can be improved by using different substrates and by increasing the substrate concentration. Moreover, this work provides insights into the first steps of substrate utilization where the methyl groups from methanol or methylamines are transferred to HS-CoM by substrate-specific methyltransferases via certain corrinoid proteins. It became clear from qRT-PCR analysis and experiments using resting cells that methanol is the preferred substrate of this organism. This leads to the question whether the constitutive expression of *mta* genes reflects the greater availability of methanol in comparison to TMA in the human gut as natural habitat of *M. luminyensis*. Interestingly, it became evident from the analysis of volatile fatty acids in human feces that methanol is indeed more abundant in the intestinal tract than methylamines while elevated TMA levels are only associated with certain diseases [44].

## Supplementary Material

Supplementary Figure 1: Dependence of methanol concentration and final optical density. Supplemntary 2: Dependence on acetate consumption and dry weight formation in the exponential growth phase of *M*. *luminyensis*.

## Figures and Tables

**Table 1:** Sequences of primer used for quantitative RT-PCR.

Gene of interest	Accession number	Sequence (5′ → 3′)
*mtaC1*	WP_019176359.1	For: ATAGATCTGAAGAGTGTA
		Rev: GCGGTAACGTCCTCGCCT
*mtaC2*	WP_019177725.1	For: TCAGACCTTGACGCAATT
		Rev: TCGGTGGTGTCATCAATG
*mtaC3*	WP_026069014.1	For: CCAGACTTTGATAATGTA
		Rev: TCAGTGACATCGTCCTGG
*mttC*	WP_026069100.1	For: CCAGATACTAGAAGACGC
		Rev: CTTCTCGCCGAATTTGC
*mtbC*	WP_026069102.1	For: GTCTGCAAGAATCGAATT
		Rev: TCCCCTTTCAAAACGAGT
*mtmC1*	WP_026068678.1	For: TGATGAAATGTTAGGACG
		Rev: CCCTTCCCGAGACCATTC
*mtmC2*	WP_026068679.1	For: AGAAGAAGTGCTGGCGT
		Rev: CCTTGGCTCAATCCGCTC
*mtmC3*	WP_049796371	For: ACAAGCTATATTGGCAGA
		Rev: CCCTTTCCCAGGCCTTGG
*mtmC4*	WP_019178518	For: GGACGAGATTCTAGCCAC
		Rev: CCCTTTCCCAGGCCGTTC
Ribosomal S15	WP_026068997	For: CTCATTCTCAGGGACCAGCA
		Rev: TGCATGTTCCTCTTGTTGGC

**Table 2:** Comparison of growth yields.

Organism	Substrate	Y_CH4_ (g cells/mol CH_4_)	Reference
*Methanogens without cytochromes*			
*Methanobacterium marburgensis*	H_2_ + CO_2_	Up to 3.0	[24]
*Methanobrevibacter arboriphilus*	H_2_ + CO_2_	Up to 2.7	[25]
*Methanobacterium bryantii*	H_2_ + CO_2_	Up to 2.5	[26]
*Methanospirillum hungatei*	H_2_ + CO_2_	Up to 1.0	[27]
*Methanosphaera stadtmanae*	Methanol + H_2_	4.0	[9]
*Methanomassiliicoccus luminyensis*	Methanol + H_2_ TMA + H_2_	2.4 2.4	This work This work

*Methanogens with cytochromes*			
*Methanosarcina barkeri*	H_2_ + CO_2_ Methanol Acetate	Up to 7.5 Up to 7.2 Up to 2.1	[24] [28] [29]
*Methanosarcina* sp. strain 227	H_2_ + CO_2_ Methanol Acetate	Up to 8.7 Up to 6.0 Up to 2.7	[30] [30] [30]

## Abbreviations

CoM-S-S-CoB: Disulfide from HS-CoM and HS-CoB
DMA: Dimethylamine
DW: Dry weight
HS-CoB: 7-Mercaptoheptanoyl threonine phosphate
HS-CoM: 2-Mercaptoethanesulfonate
M: *Methanomassiliicoccus*
MMA: Monomethylamine
MT1: Co-methyl-5-hydroxybenzimidazolylcobamide
HS-CoM: Methyltransferase
MT2, substrate: 5-Hydroxybenzimidazolylcobamide methyltransferase
Ms: *Methanosarcina*
t_d_: Doubling time
TMA: Trimethylamine
TMAO: Trimethylamine oxide
Y_CH4:_: Growth yield per mole of methane.
