# Supplementary material for: Growth Characteristics of Methanomassiliicoccus luminyensis and Expression of Methyltransferase Encoding Genes
Source: Archaea. 2017 Nov 2;2017:2756573. doi: 10.1155/2017/2756573 (PMC5688252; doi:10.1155/2017/2756573)
Supplement: Supplementary file 1 — Supplementary Figure 1: Dependence of methanol concentration and final optical density. Supplemntary 2: Dependence on acetate consumption and dry weight formation in the exponential growth phase of M. luminyensis. [file 2756573.f1.pptx]

## Slide 1
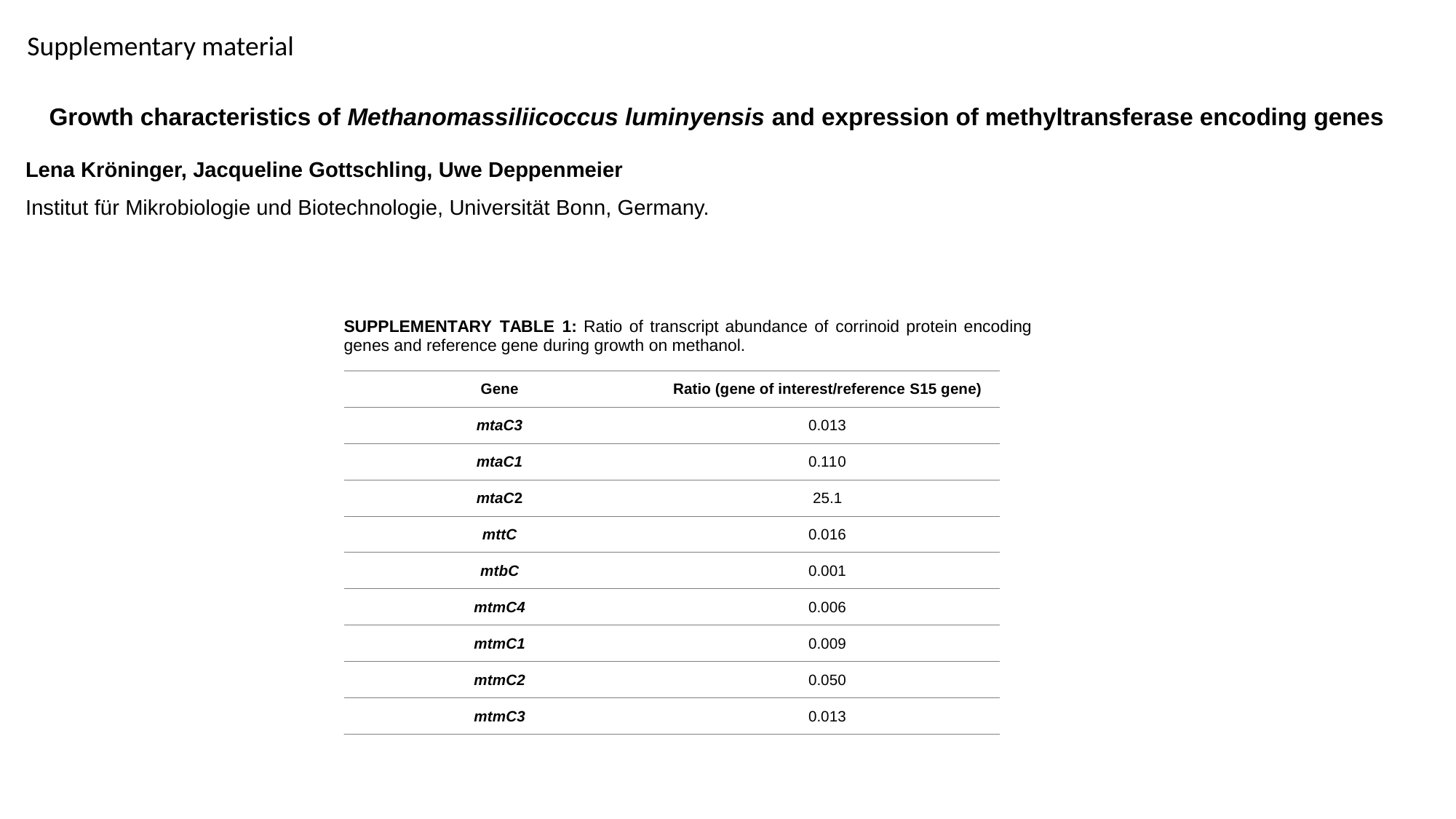

Supplementary material
Growth characteristics of Methanomassiliicoccus luminyensis and expression of methyltransferase encoding genes
Lena Kröninger, Jacqueline Gottschling, Uwe Deppenmeier
Institut für Mikrobiologie und Biotechnologie, Universität Bonn, Germany.

## Slide 2
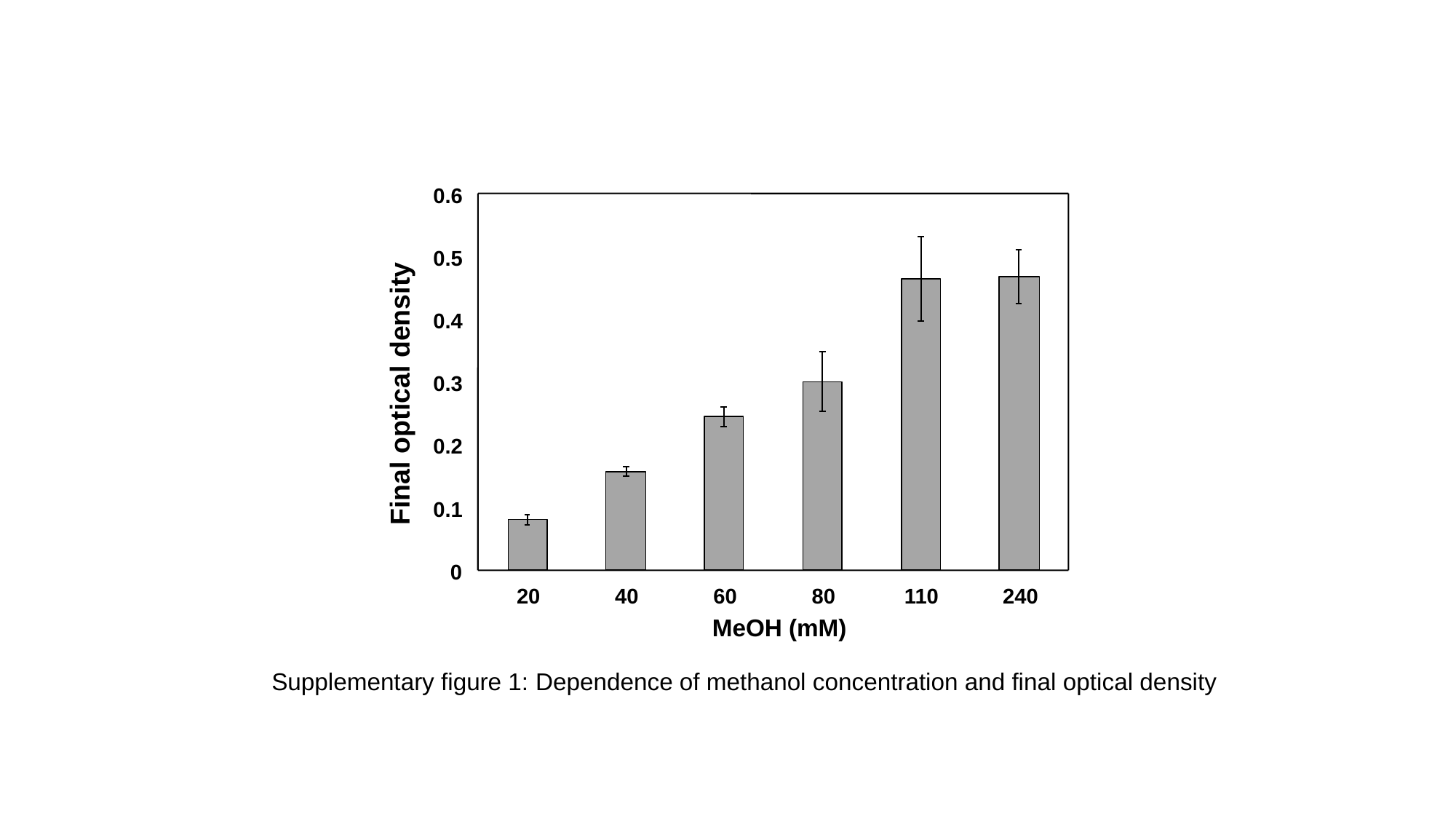

0.6
0.5
0.4
Final optical density
0.3
0.2
0.1
0
20
40
60
80
110
240
MeOH (mM)
Supplementary figure 1: Dependence of methanol concentration and final optical density

## Slide 3
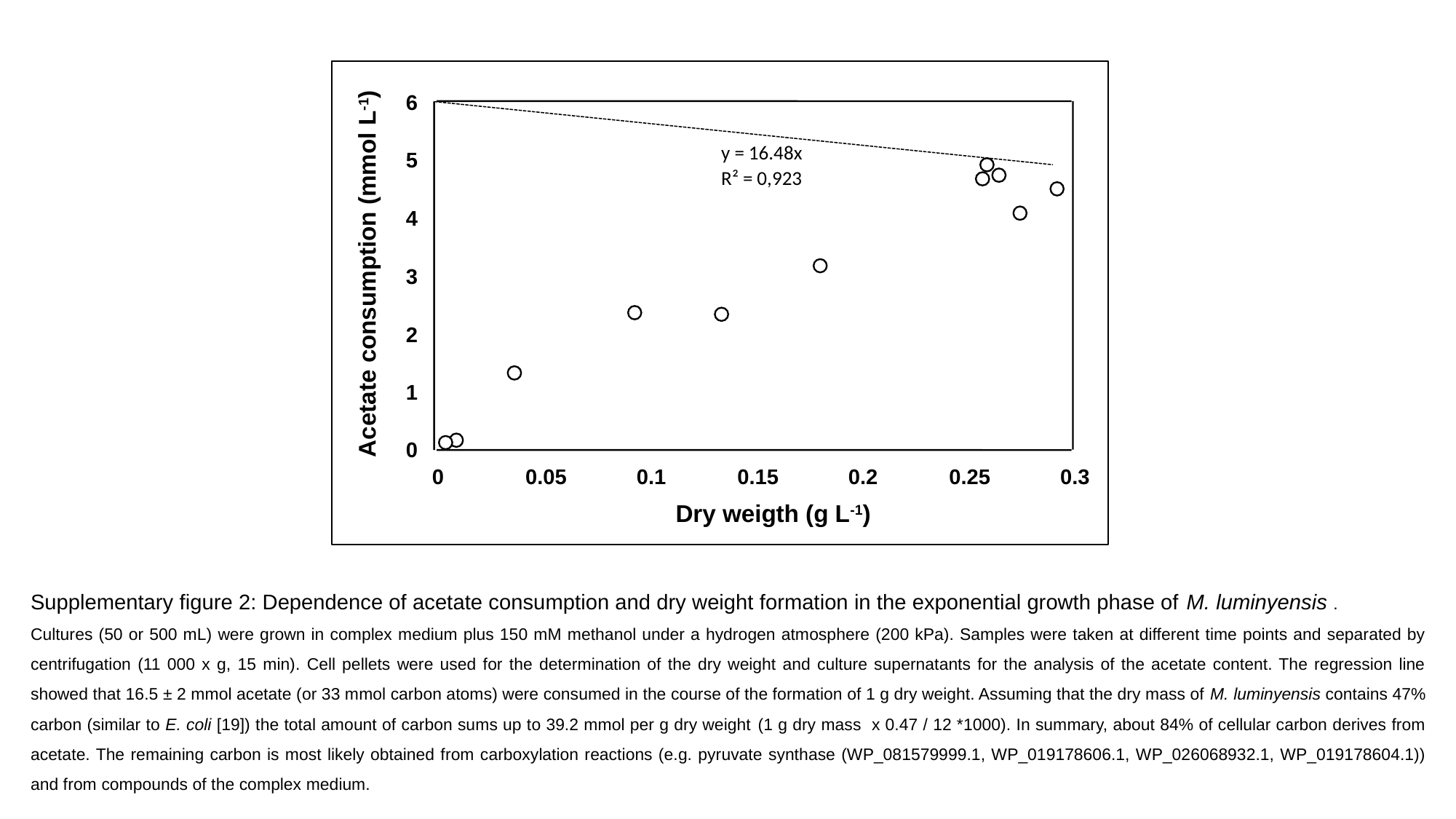

6
y = 16.48x
5
R² = 0,923
4
Acetate consumption (mmol L-1)
3
2
1
0
0
0.05
0.1
0.15
0.2
0.25
0.3
Dry weigth (g L-1)
Supplementary figure 2: Dependence of acetate consumption and dry weight formation in the exponential growth phase of M. luminyensis .
Cultures (50 or 500 mL) were grown in complex medium plus 150 mM methanol under a hydrogen atmosphere (200 kPa). Samples were taken at different time points and separated by centrifugation (11 000 x g, 15 min). Cell pellets were used for the determination of the dry weight and culture supernatants for the analysis of the acetate content. The regression line showed that 16.5 ± 2 mmol acetate (or 33 mmol carbon atoms) were consumed in the course of the formation of 1 g dry weight. Assuming that the dry mass of M. luminyensis contains 47% carbon (similar to E. coli [19]) the total amount of carbon sums up to 39.2 mmol per g dry weight (1 g dry mass x 0.47 / 12 *1000). In summary, about 84% of cellular carbon derives from acetate. The remaining carbon is most likely obtained from carboxylation reactions (e.g. pyruvate synthase (WP_081579999.1, WP_019178606.1, WP_026068932.1, WP_019178604.1)) and from compounds of the complex medium.
